# Industrial and Therapeutic Applications of Hemp: A Review

**DOI:** 10.3390/molecules31101699

**Published:** 2026-05-17

**Authors:** Harry Chiririwa

**Affiliations:** Department of Natural Sciences, Vaal University of Technology, Andries Potgieter Blvd, Vanderbijlpark 1900, South Africa; haledenc@vut.ac.za

**Keywords:** hemp, *Cannabis sativa*, industrial applications, therapeutic uses, cannabidiol (CBD), green industries, natural medicine, bioproducts, phytochemicals

## Abstract

Hemp (*Cannabis sativa* L.) is a multipurpose crop with significant industrial and therapeutic potential. This article reviews the various uses of hemp in production, building, food, cosmetics and medicine, focusing on its economic, environmental and health benefits. Industrially, hemp has been used for making fabrics, paper, bioplastics, construction materials and biofuels, because of its strong fibres, fast growth and low impact on the environment. Hemp seed oil and protein in the food and beauty industries are gaining more recognition for their nutritional and functional characteristics. Medically, compounds extracted from hemp, especially cannabidiol (CBD) and other non-psychoactive phytochemicals, have been shown to possess significant anti-inflammatory, pain-relieving, neuroprotective, antioxidant and antibacterial properties. This article talks about how better cultivation methods, processing technologies, and extraction techniques can help improve product quality, marketability, regulatory frameworks, safety standards and the quality control measures that are in place to monitor hemp production and utilization, as well as the focus on new policies in developing nations. Even though hemp has a wide range of potentials, the industry still faces difficulties in the form of laws, lack of infrastructure, unequal product standardization, and lack of scientific proof in certain areas of application. This article further identifies research gaps and points out potential areas for innovation, policymaking, and market development to be explored in the future. If backed up by proper regulations and research, hemp has great potential to contribute to the development of environmentally friendly industries, the improvement of public health and the socio-economic upliftment of communities.

## 1. Introduction

### 1.1. Background and History of Hemp Use

**Figure 1:**
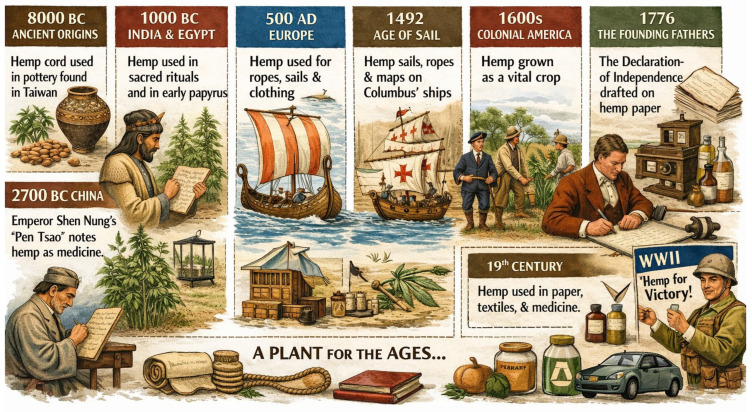
The history of hemp: a timeless plant with endless uses. *Adapted from* [7].

Hemp (*Cannabis sativa* L.) was among the earliest plants cultivated by humans [1]. Humans have been using it for fibre, food, and medicine for more than 10,000 years, as shown by archeological findings [2]. To make ropes, textiles, paper and medicinal preparations, early civilisations in China, Mesopotamia, and Egypt used hemp [3]. In ancient China, some of the first types of paper were made from hemp fibres [4]. Besides that, traditional Chinese medicine in 2700 BCE described the healing effects of hemp-based remedies [5]. Likewise, hemp was a key element in Ayurvedic medicine in India for treating pain, inflammation and digestive ailments as shown in Figure 1 [6,7].

During the Middle Ages and the Renaissance, hemp stood out as one of the key agricultural products not only in Europe but also in parts of Asia. It was extensively grown for making ropes, sails and clothes, which in turn facilitated maritime excursions and trade [8]. By the 16th and 17th centuries, the practice of hemp planting had reached the Americas, where it was promoted for both industrial and military uses. Some governments even require their citizens to grow hemp due to its critical role in making ships and fabrics. In contrast, the 20th century effectively saw a fall in hemp cultivation after the implementation of drug control measures which overlooked the distinction between industrial hemp and marijuana. These laws essentially prohibited research, marketing and general acceptance of the crop [9]. Recently, however a combination of factors such as scientific explorations, ecological advocacies and legislative changes has paved the way for hemp’s revival as an environmentally friendly plant [10]. Presently hemp is gaining more recognition for its commercial, environmental and health potential; thus, its production and use are being diversified and patented across the globe [11].

### 1.2. Legal and Regulatory Framework

The legal and regulatory framework governing hemp cultivation, processing and commercialization is of utmost importance for the development and the environmentally friendly nature of the hemp industry [12]. Even though hemp is a plant of the species *Cannabis sativa* L., it is legally distinguished from the psychoactive cannabis by its very low content of tetrahydrocannabinol (THC) [13]. In most jurisdictions industrial hemp is defined as cannabis containing less than 0.2% to 0.3% THC on a dry weight basis, the limit being set to prevent the substance from being abused and at the same time allowing farming and industrial activities that are legitimate [14]. On the one hand, drug control laws were so stringent that in addition to cannabis being categorized as an illicit drug, the production of hemp was totally restricted to the same level. As a result, scientific research was greatly restricted, investors were discouraged and the traditional hemp industries were dismantled. However, the accumulating evidence for the numerous benefits of hemp in both the economic and environmental aspects have persuaded lawmaking authorities to change the laws [15].

Therefore, governments have quantified these policies under which conditions hemp cultivation can be considered as allowed, and in such cases hemp cultivation is normally licenced and the growers are subject to inspection and compliance with the regulations. In South Africa hemp regulation is chiefly controlled by the Cannabis for Private Purposes Act and related amendments to the Medicines and Related Substances Act and Plant Improvement Act [16]. These laws set the conditions for cultivation, processing, research, and commercial distribution and authorities such as the Department of Agriculture, Land Reform and Rural Development and the South African Health Products Regulatory Authority (SAHPRA) oversee them [17]. The European Union, North America and some Asian countries have their own regulatory frameworks following the same principle, where seed certification, THC testing and traceability system compliance is mandatory [18]. Efforts have been made towards regulation but issues still exist, such as bureaucratic delays, inconsistent enforcement, limited access to finance and export regulation-related uncertainties. The alignment of international standards, clearer licencing procedures and the policy frameworks that support the sector in question are necessary elements for investment, innovation and responsible growth to be promoted in the global hemp sector [19].

### 1.3. Scope and Objectives of the Review

This review aims to provide a comprehensive overview of hemp (*Cannabis sativa* L.) industrial and medicinal uses, taking into consideration the most recent scientific studies, industry publications and regulatory documents. Essentially the review outlines hemp utilization areas such as fibre-based products, construction materials, food and nutritional supplements, cosmetics, bio-based materials and pharmaceutical and nutraceutical applications. In addition, it touches upon changes made to cultivation, processing and extraction technologies which influence the quality of products and their market use. Apart from that, the review goes into the legal, regulatory, economic and environmental aspects which either act as facilitators or barriers to the growth and sustainability of the hemp industry at the national and global level. This review is primarily a critical study of hemp and hemp-based products by referencing the existing body of literature. It also analyzes the clinical potential of major and minor bioactive constituents with a special focus on cannabidiol (CBD)/phytochemicals in hemp and safety aspects.

The review also aims to identify the reasons that hinder the large-scale adoption and hence commercialisation of hemp products. The paper also points out the untouched research areas which need to be put under a strong focus, particularly regarding issues of standardization, clinical validation and the long-term impact on the environment. The review also covers the latest innovations and market changes that might influence the future of the hemp-based industry. Grounded in a multidisciplinary synthesis of evidence, this review intends to provide a thorough understanding of the subject matter to scholars, government authorities, the industrial community and investors. It is expected that the findings of this study will be able to offer a platform for making evidence-based policy decisions, enabling sustainable development and encouraging the wise use of hemp in industries and the healthcare sector.

## 2. Botanical and Chemical Characteristics of Hemp

### 2.1. Taxonomy and Varieties of Cannabis sativa

**Figure 2:**
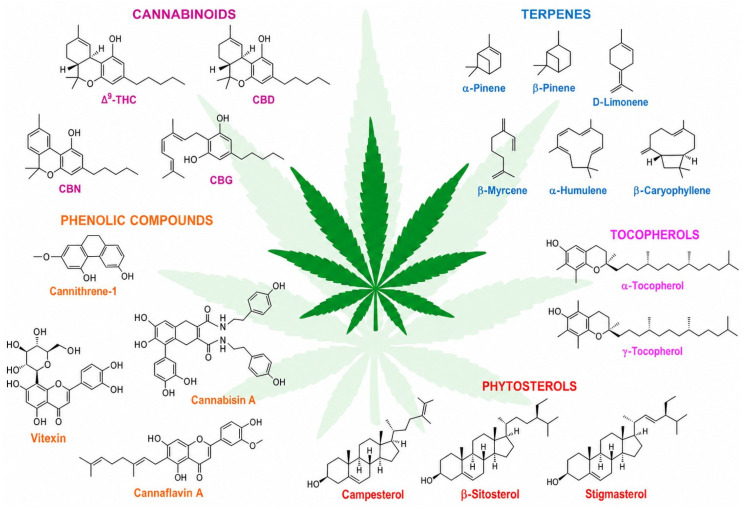
*Cannabis sativa* subsp. sativa phytochemicals [27].

*Cannabis sativa* L. is a species of the family Cannabaceae that is an annual, dioecious flowering plant widely cultivated for its industrial, nutritional and medicinal uses [20]. The genus Cannabis has been a subject of taxonomic dispute for a long time due to its high morphological variability and its tendency to hybridize extensively [21]. The three main species were traditionally thought to be: *Cannabis sativa*, *Cannabis indica* and *Cannabis ruderalis* [22]. Nowadays however, many taxonomists consider Cannabis to be a monotypic genus, one single species (*Cannabis sativa* L.) with different subspecies, varieties and cultivars adapted to various uses and environments [22]. Hence, according to this classification, *C. sativa* subsp. Sativa (Figure 2) refers to industrial hemp varieties with low levels of tetrahydrocannabinol (THC) and high fibre and seed yield [23]. These cultivars are primarily cultivated for textile production, construction materials, food products and bio-based industries. Meanwhile, *C. sativa* subsp. indica usually contains higher levels of psychoactive compounds and is mainly grown for medicinal and recreational purposes [24,25]. The trait which sets apart *Cannabis ruderalis* is its auto flowering characteristic, and it is mainly utilized in breeding programs to develop hardy hybrid varieties [26].

Through modern breeding methods, genetically stable hemp cultivars have been developed in large numbers and certified. These cultivars have been uniformly characterized for growth and have been found to be following the THC concentration limits of the law. Such varieties are differentiated by the various traits the breeders select including the quality of the fibre, fatty oil in seeds, cannabinoid composition, resistance to diseases and suitability to the environment [28]. Some of the names recognized in Europe and other parts of the world are Futura 75, Fedora 17, Carmagnola and Kompolti [29]. Recent developments in molecular genetics and genome analyses have facilitated better classification and identification of Cannabis strains; thus, breeders and quality controllers can do their work with more accuracy [30]. The knowledge of the taxonomy and genetic variation in *Cannabis sativa* is not only necessary for improving cultivation techniques but also for meeting the requirements of the law and providing a solid base for the sustainable development of the hemp industry.

### 2.2. Phytochemical Profile (Cannabinoids, Terpenes, and Flavonoids)

Hemp (*Cannabis sativa* L.) contains various bioactive compounds that have industrial, nutritional and therapeutic value. The major groups of phytochemicals that are found in hemp are cannabinoids, terpenes and flavonoids, which serve different biological and functional purposes. Genetics, growing environment, harvest time and post-harvest processing techniques all affect the makeup and level of these substances [31].

#### 2.2.1. Cannabinoids

Cannabinoids are a group of chemical compounds that occur only in the Cannabis genus. Currently, more than 120 cannabinoids have been isolated, with cannabinoid (CBD) being the most abundant compound in industrial hemp. CBD is different from tetrahydrocannabinol (THC) in that it does not cause a psychoactive effect [32,33]. CBD has been shown to have anti-inflammatory, analgesic, anxiolytic, neuroprotective and antioxidant properties [34]. In addition, cannabigerol (CBG), cannabichromene (CBC), cannabinol (CBN), and cannabidiolic acid (CBDA) are other cannabinoids that have been found in hemp and have therapeutic potential [35]. THC is strictly limited to low levels in industrial hemp strains, usually below 0.2–0.3%, thus ensuring that these products are compliant with legal standards.

#### 2.2.2. Terpenes

Terpenes are a class of volatile substances that have the powerful scent and flavour responsible for giving hemp its unique smell and taste. They play a role in the plant’s pharmacological properties through synergistic interactions with cannabinoids which are termed the “entourage effect” [36]. Some of the major terpenes in hemp are myrcene, limonene, pinene, caryophyllene, linalool and humulene. These substances have been found to display antimicrobial, anti-inflammatory, anxiolytic and antioxidant activities [37]. Besides being important as medicinal compounds, terpenes are also extensively used in the cosmetics, food and fragrance industries [38].

#### 2.2.3. Flavonoids

Flavonoids are a type of polyphenolic compound, which have a part in the colouring, UV protection and defence systems of hemp plants. Apart from common plant flavonoids such as quercetin, kaempferol, luteolin and apigenin, hemp also has a very special class of flavonoids called cannflavins (cannflavin A, B, and C) [39]. Cannflavins have been found to have very strong anti-inflammatory and antioxidant effects and might be more effective than regular non-steroidal anti-inflammatory drugs [40]. Flavonoids increase the nutritional and medicinal benefits of hemp-based products [41].

### 2.3. Synergistic Interactions and Functional Significance

The overall biological activity and functional properties of hemp are due to the combined action of cannabinoids, terpenes and flavonoids. The complex interaction of these phytochemicals increases the therapeutic efficacy of the products, making them more stable and thus giving them a wide range of industrial applications [42]. For standardization, quality control and the creation of high-value pharmaceutical, nutraceutical and cosmetic products, it is very important to understand the phytochemical profile of hemp.

### 2.4. Fibre and Biomass Composition

Hemp (*Cannabis sativa* L.) is mainly known for its great quality fibre and biomass that are the basis of its industrial uses. The plant is made up of two major parts: the long, strong bast fibres on the outside and the woody, very light inner core, which is called hurds or shives. The combination of these parts makes hemp a strong, durable and versatile material for the use of different industries like manufacturing and construction [43].

#### 2.4.1. Bast Fibres

Bast fibres are situated at the outermost layer of the hemp stem. They are very long, strong (in tensile strength) and flexible. The main constituents of these fibres include cellulose (around 55–72%), hemicellulose (8–19%), and lignin (2–5%), with the remainder being pectin, waxes and proteins. The elevated cellulose level renders hemp fibres advantageous for making textiles, ropes, and composite materials and producing high-quality insulation materials. When compared to cotton and synthetic fibres, hemp bast fibres have better resistance to ultraviolet light, microbial attack and mechanical abrasion; thus, they can be used for a longer time [44].

#### 2.4.2. Woody Core (Hurds/Shives)

The central part of the hemp stem is essentially made up of short porous fibres that have a high content of lignin (20–30%) and cellulose (30–40%). Such a microstructure gives the material very good absorbency and heat insulation and allows the lightness of the product to be retained. Hemp hurds find a wide range of applications such as hempcrete, animal bedding, particle boards, paper products and biodegradable packaging materials. Their excellent porosity property acts as a moisture moderator when used in the building sector, which in turn results in better indoor air quality and increased structural longevity [45].

#### 2.4.3. Whole Plant Biomass Composition

In addition to fibres, hemp generates biomass from the leaves, flowers, seeds and roots. The leaves and flowers have a high concentration of cannabinoids, terpenes and phenolic compounds and are very useful in the pharmaceutical industry and for the extraction of nutraceuticals. Hemp seeds are made up of approximately 25–35% oil and 20–25% protein and have a very healthy fatty acid profile with omega 3 and omega 6 fatty acids being the most dominant. The leftover biomass such as stalks and processing by-products can be used for bioenergy production, composting and soil enrichment [46].

#### 2.4.4. Industrial Significance

The balanced combination of fibre and biomass makes hemp a green raw material and its fast growth, ability to capture large amounts of carbon and hardly any need for agrochemicals make it more ecological. It is used in an efficient manner and the environmental impact of agriculture is lowered. The circular economy is supported by the rational use of all components of a plant and economic returns are maximized. Therefore, knowing fibre and biomass composition plays a major role in leveraging processing technologies, enhancing product quality, and uplifting hemp’s industrial applications [47].

## 3. Cultivation and Agronomic Practices

### 3.1. Climatic and Soil Requirements

The successful cultivation of hemp (*Cannabis sativa* L.) largely depends on climatic conditions and soil characteristics, which ultimately affect plant growth, fibre quality, biomass yield and phytochemical composition. Hemp is a quick-growing and versatile plant that can flourish in temperate to subtropical climates and has its optimum physiological condition under moderate environment [48].

#### 3.1.1. Climatic Requirements

Hemp needs a warm season with plenty of sunshine and moderate rainfall to grow. It grows best in temperatures between 15 °C and 27 °C, but it can endure a few days at a higher temperature. The seed starts to grow when the soil temperature is about 8–10 °C and the plant’s development and flowering are accelerated by longer daylight hours. Hemp is a photoperiod-sensitive plant; therefore, its flowering starts when the day length decreases generally below 14 h. Annual precipitation requirements are between 500 and 750 mm, and this amount needs to be evenly distributed during the growing season. Adequate moisture is highly important especially during the early vegetative and flowering stages. Extended periods of drought may lead to a decrease in fibre yield, seed production and cannabinoid levels, whereas too much rain may result in fungal diseases and root rot. In areas where rainfall is not regular, additional irrigation is advised to achieve stable crop results [43].

#### 3.1.2. Soil Requirements

Hemp thrives most when it is grown in well-drained fertile soil that has good water holding capacity. A mixture of loamy and sandy loam soil which is rich in organic matter is considered perfect, as it helps the roots to become very strong and nutrients to be absorbed easier. Generally clay soils that are heavy and have poor drainage are not suitable for growing hemp because they can cause waterlogging and the roots may not get enough oxygen. The best soil pH for growing hemp is between 6.0 and 7.5. Slightly acidic to neutral environments are the best conditions for nutrients to become available to the plants and for soil microorganisms to be active. Hemp needs to have a sufficient supply of essential nutrients especially nitrogen, phosphorus, potassium, calcium and magnesium. Of these nutrients, nitrogen is very crucial during the periods when the plants are just starting to grow rapidly [49].

#### 3.1.3. Adaptability and Regional Considerations

Hemp displays a great ability to adjust to different agro-ecological zones and can be grown in many parts of the world, including southern Africa. However, factors such as local climate changes, soil nutrient level and water availability should be closely checked for an increase in output and quality. Proper site selection and soil management practices are necessary to ensure sustainable hemp production [50].

### 3.2. Cultivation Techniques

Good cultivation practices are necessary if we want to get maximum productivity, quality and sustainability from hemp (*Cannabis sativa* L.). Suitable agronomic practices are among the main factors determining the amount of fibre and seed yield and the level of phytochemical content, thus allowing the exploitation of the industrial and therapeutic potentials of the plant [51].

#### 3.2.1. Land Preparation

To get even germination and quick hemp growth, seedbeds must be very well prepared. It is best to do deep ploughing to break the hard layer of soil and make it easy for the roots to go down. After that, do harrowing to get the seedbed’s smooth, soft and level surface. It is advisable to weed and clean crop residues and stones from the field before planting. This helps to reduce the competition of the plants and make it easier for the mechanical harvesting [52].

#### 3.2.2. Seed Selection and Sowing

The choice of certified top-notch seeds that are perfectly matched with local climatic conditions is very important for the success of cultivations. Different types of cultivars are utilized depending on whether the purpose is to produce fibre, seed or cannabinoids and farmers must select the right varieties based on the end-use as well as the regulations. The time for sowing is generally spring or early summer when the temperature of the soil reaches more than 8 °C. In the case of fibre production, very dense planting (60–100 kg/ha) is advised to produce tall straight stalks with very few branches. For seed and cannabinoid production, it is better to use a lower density (20–30 kg/ha) to allow the plants to branch and produce flowers. Normally the seeds are set at a depth of 23 cm to make sure that the plants emerge quickly [53].

#### 3.2.3. Nutrient Management

Hemp is a crop that has a high demand for nutrients and needs fertilization in a balanced way. Nitrogen is crucial for the growth of the vegetative part, whereas phosphorus helps the roots get stronger and the plant to flower, and potassium raises the resistance of the plant to diseases and improves the quality of the fibre. Organic materials like compost and manure help to improve the soil structure and release nutrients into the soil. Soil analysis/residue examination should be conducted before planting to have a proper fertilization program and avoid nutrient imbalances [54].

#### 3.2.4. Water Management

There is need for a considerable amount of water in the crop at germination, early growth and flowering. Normally hemp requires some 500–750 mm of water throughout the growing season but this varies according to the climate and to soil conditions. For locations with extremely erratic rains, growers rely on drip or sprinkler irrigation. Over-watering should be avoided as too much moisture in the soil will rot the roots and cause diseases [55].

#### 3.2.5. Weed, Pest and Disease Management

Hemp shows vigorous early growth and canopy formation that inhibit weeds from growing, and controlling weeds is necessary during the very first stage of establishment. Methods like mechanized weeding and crop rotation are mainly utilized in organic and sustainable systems (Table 1).

Although hemp is a hardy plant and can resist most pest and disease attacks, it can still be vulnerable to aphids, mites, fungi and bacteria. To keep the environment healthy and the products safe, it is best to use Integrated Pest Management (IPM) methods such as biological control, resistant varieties and the very limited use of only approved pesticides [56].

#### 3.2.6. Harvesting and Post-Harvest Handling

The method of harvesting depends on what the crop will be used for. Fibre hemp is usually harvested at the early flowering stage to get the best quality of the fibre while seed and cannabinoid hemp are harvested when fully mature. For large-scale production, mechanized harvesters are generally used. After harvest, the crop must be dried, retted (if fibre), cleaned and stored in controlled conditions to inhibit mould growth and maintain quality. Proper handling guarantees the continuation of fibre strength, seed viability and phytochemical integrity [57].

#### 3.2.7. Sustainable Cultivation Practices

Sustainable hemp farming is about protecting the land soil, making good use of water and safeguarding the diversity of life. Crop rotation, less ploughing, and using organic fertilizers and precision farming tech are some of the ways that help to keep the land productive for a long time and environmentally sustain good hemp crops [58].

## 4. Industrial Applications of Hemp

### 4.1. Textile and Fibre Industry

**Figure 3:**
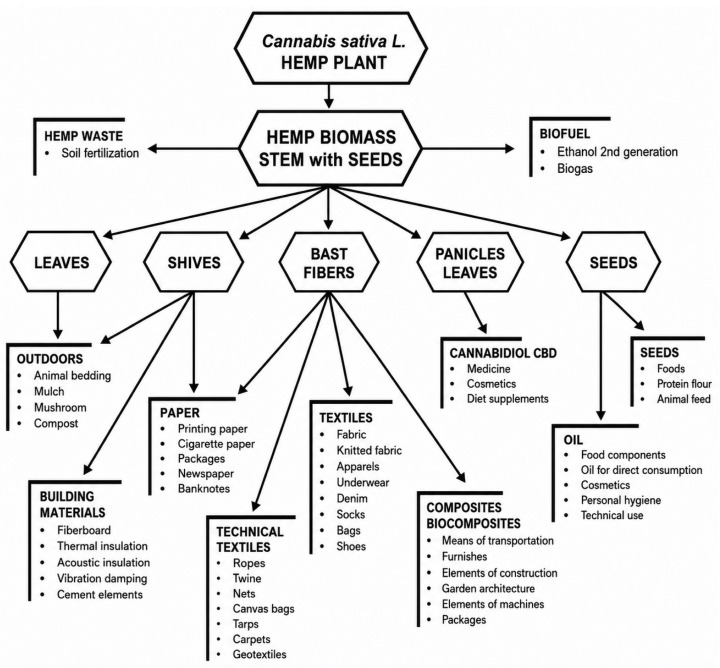
Modern uses of industrial hemp plant raw material, (CC BY 4.0) [60].

Hemp (*Cannabis sativa* L.) is a natural fibre stock which is probably one of the oldest and most adaptable. It is widely acclaimed for its strength, durability and for being environmentally friendly. Hemp fibres for making textiles come from the bast layer of the plant stem and are known for their high tensile strength, for being very resistant to abrasion and for having excellent moisture absorption properties. These qualities from the very nature of the material influence hemp to become a highly desirable raw material for textile and industrial fibre applications (Figure 3).

For centuries, hemp fibres have been utilized in making ropes, sails, canvas and sacks because of their excellent mechanical properties. Recently the development of fibre processing techniques like enzymatic retting, steam explosion and mechanical decortication have resulted in the production of fibres that are not only finer but also softer and thus suitable for high-quality apparel and household textiles. Nowadays hemp textiles are constantly finding their way in the apparel industry, as well as in upholstery, carpet and industrial applications [59].

Between its antimicrobial, UV resistance and thermoregulatory properties, hemp fibre has been demonstrating that it is quite comfortable to wear and that the product maintenance is relatively long. On a comparative note, cotton and synthetic fibre production is more water- and chemical-intensive than that of hemp, which largely has contributed to a reduction in environmental impact. Moreover, hemp has a positive influence on soil by keeping its structure intact and preventing it from erosion, so sustainable agriculture is also supported in this way. In line with industrial use, hemp fibres have been massively incorporated in the production of geo-textiles and insulation materials and as the reinforcing elements of composites for automotive and construction uses. Composites based on hemp have become a great solution as they are kinder to the environment, lighter in weight and stronger than glass fibres and synthetic polymers.

However the benefits are just a few as issues such as fibre inconsistency and the lack of vast processing facilities and standardization are still unaddressed. More funding in technology developments for processing, the introduction of tighter quality control measures and the evolution of the supply chain is essential if hemp textiles are to be a dominant force in global markets. The textile and fibre industry can be a great source of value addition and a leading industrial development mode within the hemp sector [61].

### 4.2. Construction Materials (Hempcrete and Insulation)

One of the foremost hemp-based construction materials mentioned is hempcrete while another one is hemp fibre insulation. They have been getting a lot of attention lately among sustainable and environmentally friendly alternatives to conventional building materials. Hempcrete is a bio-composite made of hemp core material (the woody parts of hemp plant known as hemp hurds or shives) mixed with lime-based binder and water. The resulting product has low density, is permeable to air and is green, which means that it is usable in walls as well as floors or roofs. Hempcrete is a very good thermal insulator because it has many tiny air pores which hinder heat flow, and at the same time it maintains a comfortable temperature inside the house. It can also effectively insulate a space from noise which is a contributing factor towards a better standard of living. Hempcrete has very high vapour permeability and moisture can easily evaporate through the wall; hence, the problem of condensation, mould and material decay is significantly reduced. All these aspects directly or indirectly contribute to a healthier home and a longer life of the building. One of the main points that hempcrete is often praised for is that it has a very minimal environmental impact. What goes into the making of hempcrete is a small fraction of what a house requires in terms of natural resources and energy consumption. Moreover, hemp through photosynthesis takes up a lot of carbon dioxide from the atmosphere while at the same time carbon dioxide is slowly reabsorbed by the lime binder through carbonation. This twofold carbon trapping enables hempcrete to be either carbon negative or carbon neutral over the whole life cycle of the material [62].

Compared to conventional concrete and synthetic insulation materials, hemp-based products still have an advantage over them in that they use less energy in production and produce less greenhouse gases. Hemp fibre insulation is made from the processed bast fibres that are bonded using natural or low toxicity binders. These insulation mats are not only renewable and biodegradable but also free from harmful volatile organic compounds, which makes them safer both for the installers and the occupants. They can be highly resistant to pests, fungi, and fire if properly treated; hence, the product has enhanced durability and safety. However, there are still several factors that limit the widespread use of hemp-based construction materials. These factors include regulatory barriers, a lack of standardized building codes, and limited technical knowledge in some areas [63]. More research, certification, and policy support is needed to help the market grow and the products to become integrated into mainstream construction practices. Hempcrete and hemp insulation are great innovations that can contribute to making building systems sustainable, energy-efficient and climate-resilient [64].

### 4.3. Paper and Packaging Products

Hemp as a natural plant fibre with the highest content of cellulose has been one of the most used raw materials for paper production since the advent of paper making. The use of hemp fibre for paper making can be traced back to the eastern world, especially in areas such as China, Korea and Japan, in which hemp fibre paper represented a large proportion of the paper stock. In China there were paper mills capable of turning out paper products of hemp fibre, flax fibre and rags by the year 62 AD. Industrial hemp production is increasing rapidly due to the increased demand for sustainable and biodegradable products. Hemp bio-composite is widely used for automotive parts, interior panels and other consumer goods. Hemp paper is very durable and tear-resistant, and hemp fibre paper has twice the tear strength and four times the tensile strength of wood fibre paper. Hemp fibre paper can be re-used and recycled for a longer period, making it very eco-friendly. Hemp paper manufacturing requires less energy consumption and water resources and produces fewer wastewater pollutants due to a reduced use of chemicals and bleaching agents as compared to wood-based paper production. These benefits certainly mitigate the adverse environmental effects of the paper and packaging industries and contribute to the sustainable development of these businesses [65].

When hemp fibres are combined with biopolymers or recycled materials, they not only improve structural integrity but also keep the whole environmental aspect sustainable. Moreover, it has historically been a small feature that hemp-derived nanocellulose and microfibrillated cellulose have been used principally for the packaging industry of advanced systems. These materials have been shown to substantially increase the barrier properties of packaging against oxygen, moisture and grease, which indirectly helps in extending the shelf life of the product and in turn reduces food waste. Hemp-based packaging materials hold the promise of being utilized for antimicrobial and active packaging purposes too once they are coupled with natural extracts. Slow and difficult moves towards the incorporation of hemp on a large scale in the paper and packaging sector are due to a lack of adequate processing facilities, higher starting production costs, and strong competition from the currently well-established wood pulp industries. There is an ongoing demand for continuous technological breakthrough, investment and policy support to keep the cost efficiency level high and thus encourage market competitiveness. Hemp-based paper and packaging products could be a part of environmentally friendly solutions while also supporting circular economy concepts [66].

### 4.4. Automotive and Bio-Composite Industry

Hemp fibres are getting increasingly recognized and thus attracting significant attention in the automotive and bio-composite domain applications as green alternatives to the tradition synthetic and mineral reinforcements. Being of low density, high specific strength, biodegradable and renewable, hemp fibres are progressively getting into the production of vehicle components made of lightweight composite materials. Such materials enable the vehicle to be lighter; therefore, the vehicle consumes less fuel and emits less greenhouse gases. Hemp-based bio-composites are essentially used in making car interiors parts like door panels, dashboards, the back of seats, headliners, parcel shelves and trunk liners. When upped with either thermoplastic or thermosetting polymer, mechanical strength, impact resistance and vibration-damping properties can be significantly improved by hemp fibre. Their natural acoustic insulation property helps in enhancing the reduction in the noise level inside the car and thus keeps passengers comfortable. These days big car manufacturers are using natural fibre composites in their sustainability and lightweighting initiatives [67].

Generally hemp bio-composites are made through frequently employed processes such as compression moulding, injection moulding, resin transfer moulding and extrusion. Thus these methods allow for large-scale production in the most efficient way, and the material is always of a consistent quality. Advancements in surface modification, chemical treatment and fibre hybridization have recently made it possible to enhance fibrematrix compatibility, durability and moisture resistance, which was a main problem hindering the use of natural fibre composites in the past. Outside the automotive industry, hemp-based bio-composites are becoming even more popular for making furniture, consumer goods, sporting equipment and construction panels. These materials deliver good life cycle performance, reduce carbon footprints, and can be recycled more easily in comparison with the conventional composites reinforced with glass or carbon fibres. Besides these benefits, hemp fibres are also more user-friendly, generating fewer airborne particles and lessening the health risks during manufacturing [68].

Nevertheless, there are difficulties that persist such as the changing quality of the fibre, its hygroscopic nature and the lack of sufficient performance data over the long term under severe environmental conditions. To facilitate market acceptance there needs to be standardization of processing methods, better fibre treatment techniques and more studies on durability. The automotive and bio-composite industry is a very promising sector for the application of hemp as it supports sustainable manufacturing and the circular economy.

### 4.5. Biofuels and Bioplastics

Industrial hemp is being regarded increasingly as a multi-use and eco-friendly raw material that can produce biofuels and bioplastics; hence it is facilitating the world to move towards renewable energy and green materials. Thanks to its enormous yield, rapid growth and an ability to grow well in practically any climate, hemp is a wonderful source of energy and polymer material production. In the biofuel sector, hemp plant matter can be transformed into bioethanol, biodiesel, and biogas through biochemical and thermochemical processes. The cellulose and hemicellulose inside the hemp stalks can be directly fermented to bioethanol after pretreatment and enzyme hydrolysis. Hemp seeds contain oils that may be converted to biodiesel through the trans-esterification method, while the remainder of the raw material is available for biogas production by using anaerobic digestion or for bio-oil making through pyrolysis. These biofuels have significantly lower GHG emissions and provide a better energy supply than fossil fuels [69].

Hemp-derived cellulose, lignin and oils are a very attractive source of raw materials for bioplastics as well. If cellulose from hemp fibres is properly extracted, it can be used to make bio-degradable polymers, films and packaging materials. Hemp seed oil can be a renewable plasticizer and polymer precursor. Hemp fibres when blended with biopolymers like polylactic acid (PLA) and polyhydroxyalkanoates (PHA) play a major role in improving the mechanical, thermal and longevity properties of bioplastic products. Numerous research work focusing on hemp nanocellulose and bio-based resins have been made with the aim of producing high-quality bioplastic applications such as flexible electronics, medical devices and eco-friendly packaging. This combination makes the products have superior barrier properties, recyclability and functional performance. Hemp bioplastics present an eco-friendlier choice since they are biodegradable and compostable in some cases, thus helping to decrease plastic pollution and lessen the pace at which plastic accumulates in the environment [70].

Although they have great potential, the large-scale commercialization of hemp-based biofuels and bioplastics is still limited to relatively small amounts because of the elevated processing costs, technological constraints and competition from well entrenched petrochemical industries. Investments in biorefinery technology, supply chain integration and regulatory support are necessary to increase profitability and viability. Hemp-based biofuels and bioplastics could be viable options for sustainable industrial development and circular economy implementation.

### 4.6. Food and Nutritional Products

Hemp seeds and their products are getting more recognition as nutritional powerhouses in a variety of applications for human consumption. Hemp seeds have a complete profile of essential amino acids along with a large amount polyunsaturated fatty acids (PUFAs), dietary fibre, vitamins and minerals, thus making them an excellent and plant-based source of protein as well as nutrients. The seeds have a great amount of omega 3 and omega 6 fatty acids in the right ratios to each other which is good for the heart, brain function and anti-inflammatory response facilitation [71].

Hemp seed oil is in demand as a supplement and as a functional food ingredient largely due to its richness in linoleic acid, alpha linolenic acid and other components like tocopherols and phytosterols that are very beneficial to health. It can be used to make salad dressings, smoothies and fortified foods, giving foods added nutritional and health value. Protein powder not only serves the consumer looking for plant-based protein but also contributes to environmental sustainability. Apart from seeds and oil, hemp leaves and flowers are regarded as potential functional food ingredient sources which deliver flavonoids, polyphenols and a small number of cannabinoids with antioxidant and anti-inflammatory activities. The marketplace is getting new hemp-based drinks, snacks and nutraceuticals that attract the attention of health-conscious consumers who are looking for plant-derived functional foods [72].

Hemp’s versatility, rapid growth, and low requirement for agrochemicals justify it as a sustainable crop for large-scale food production. Adding hemp to the diet helps to ensure nutritional security, provides variety in protein sources, and brings functional health benefits. On the other hand, issues related to regulations, allergens, and public perception need to be solved before hemp-based foods can be widely distributed worldwide.

### 4.7. Cosmetics and Personal Care Products

The cosmetics and personal care industry has shown a great deal of interest in hemp and all its derivatives. This is primarily because of their ability to moisturize, reduce inflammation, and act as antioxidants, and their skin-nourishing characteristics. Hemp seed oil, which is packed with essential omega fatty acids, vitamins (A, D, and E) as well as phytosterols, has become a staple ingredient in skin creams, lotions, serums and lip care products. The oil’s properties as an emollient assist in keeping the skin barrier intact, thus facilitating skin hydration and preventing water loss through the skin. This makes it perfect for people with dry, sensitive or ageing skin. The cannabinoids, with cannabidiol (CBD) at the forefront, are used in topical preparations owing to their anti-inflammatory, analgesic and antioxidant properties. CBD-containing creams, balms and oils have demonstrated a significant ability in decreasing skin irritation, redness and pain that come with disorders of the skin such as eczema, psoriasis and acne. Hemp-derived flavonoids and terpenes work to increase the antioxidant and calming effects, thereby shielding the skin from damage through oxidative stress and environmental factors [73].

Besides skin care, hemp oils and extracts are also being utilized in the production of hair care products such as shampoos, conditioners and hair masks. These products work towards hair strength as well as moisture retention and scalp rejuvenation. Hemp can be used as a bio-based sustainable substitute for synthetic elements in the formulation of cosmetics because of its chemical structure. This satisfies the demand from the consumers who are increasingly going green and are also against animal testing [31].

Challenges such as standardizing active ingredients and ensuring product stability and compliance with regulations still pose major hurdles for the mass marketing of these products. However, hemp-based cosmetics can become a pool of innovations in natural, functional, and eco-friendly personal care products.

## 5. Therapeutic and Medicinal Applications of Hemp

**Figure 4:**
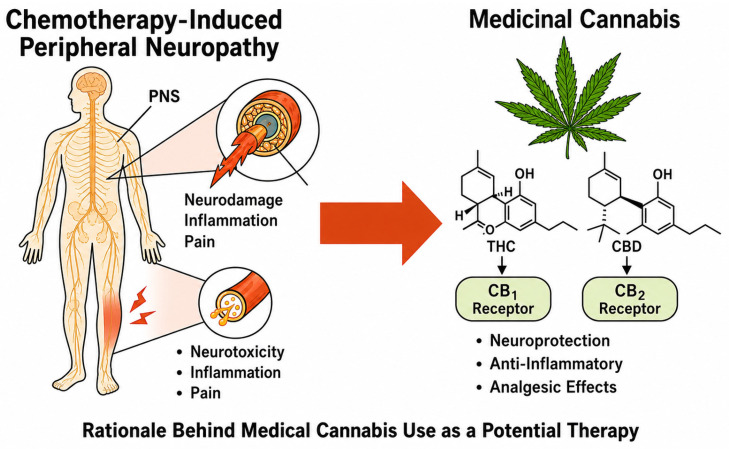
Potential therapeutic targets of THC and CBD via CB1/CB2 receptors [76].

Industrial hemp is characterized by a rich phytochemical profile of phytochemicals, such as cannabinoids, terpenes, flavonoids, and polyphenols (Figure 4), which endow it with various medicinal properties [31]. In contrast to marijuana, hemp has only trace amounts of tetrahydrocannabinol (THC) which is responsible for the “high”. Therefore, it can be used for therapeutic purposes without causing mind-altering effects [74]. A growing body of research has paved the way for the manufacture of hemp-based drugs, health supplements, and general health products.

### 5.1. Cannabidiol (CBD) and Non-Psychoactive Compounds

Cannabidiol (CBD) is the major non-psychoactive cannabinoid in *Cannabis sativa* L that has become the main target for the therapeutic, medicinal, and wellness uses of hemp. CBD will not make you “high” or alter your mind, unlike 9-tetrahydrocannabinol (THC), so it is safe for use by people of all ages, including kids and seniors. Activation of the endocannabinoid system by CBD, mostly through the indirect modulation of CB1 and CB2 receptors (Figure 4), affects several physiological processes such as neurotransmitter release, immune response and cellular signalling pathways [75].

Besides cannabidiol (CBD), hemp is packed with many other non-psychoactive compounds, like minor cannabinoids such as cannabigerol (CBG), cannabichromene (CBC), and cannabinol (CBN) in addition to terpenes and flavonoids. These compounds give hemp its medicinal characteristics by synergistic interactions resulting in the enhancement of anti-inflammatory, pain-relieving, anxiety-reducing and neuroprotective effects [77]. Good examples of terpenes include myrcene, limonene and caryophyllene which regulate the activity of cannabinoids, increase the rate of their absorption and have antioxidant and antimicrobial properties. Multi-compound hemp extracts containing various non-psychoactive substances align well with the entourage effect concept, suggesting that full-spectrum extracts may be more therapeutically potent than using isolated CBD only [78]. Consumers can find non-psychoactive cannabinoids and other plant chemicals being used more in oil, capsule, topical, beverages and nutraceutical formulations, thereby providing safe and versatile alternatives for therapeutic, cosmetic and wellness needs. Standardization, quality control and accurate labelling are indispensable if these products are to be safe, reliable and efficacious in a reproducible manner.

### 5.2. Cannabigerol (CBG)

Cannabigerol (CBG) is an unaltered cannabinoid in *Cannabis sativa* L and is commonly called the mother cannabinoid since it is the precursor of other cannabinoids such as THC, CBD and CBC. In contrast to THC, CBG has no mind-altering effects and can be used for therapeutic and wellness purposes. CBG attaches to the endocannabinoid system as it binds to CB1 and CB2 receptors and influences various signalling pathways, among them the ones related to inflammation, neuroprotection and cellular homeostasis [79].

Studies indicate that CBG has a wide range of beneficial effects on the body such as anti-inflammatory, antibacterial, neuroprotective, and anticancer properties. It may be useful in lowering the pressure inside the eye in glaucoma, calming inflammatory bowel disease, and preventing the growth of some bacterial strains, including antibiotic-resistant Staphylococcus aureus. Furthermore, CBG may aid in appetite control and neurons’ protection, which further highlights its use for neurodegenerative conditions like Huntington’s and Parkinson’s diseases [80].

Normally, CBG is found in hemp at very low concentrations which in the past has limited its commercial availability. Selective breeding, extraction techniques, and cultivation of CBG-rich chemotypes have been the major tools to increase production, thus allowing the incorporation of CBG into oils, tinctures, topicals, and nutraceutical formulations [81]. The soothing attributes of CBG together with its non-psychoactive nature make it a potential compound which will probably be researched and developed as a safe, plant-based medicinal product in the future.

### 5.3. Cannabichromene (CBC)

Cannabichromene (CBC) is a cannabinoid with no psychoactive properties isolated from *Cannabis sativa* L which is normally present at lower levels than CBD and THC. Along with other non-psychoactive cannabinoids, CBC does not induce euphoria and is mainly considered for its health benefits. It mainly affects the endocannabinoid system in an indirect way, thus affecting cannabinoid receptors and increasing the effectiveness of other cannabinoids through the entourage effect [82]. Besides the anti-inflammatory and analgesic effects demonstrated by CBC, it also has antimicrobial and neuroprotective properties [83]. Animal and cell model studies provide evidence that CBC can facilitate the growth of new brain cells, diminish gut inflammation, and when used in combination with CBD and THC [84], it may further enhance analgesic and antidepressant effects [85]. The bacteriostatic nature of CBC may also be an excellent attribute for the topical application of skin-care products, especially for acne [86].

Hemp contains only a very small amount of CBC naturally, so CBC is usually co- extracted with other cannabinoids from the full spectrum to get the maximum therapeutic effect. Scientists have been conducting various studies on CBC, and there is a rising interest in its potential ability to counteract disease symptoms such as pain, inflammation, neurological disorders, and skin conditions. The presence of CBC in cannabinoid oils, tinctures and skin-care products demonstrates its key role as a complementary non-psychoactive compound in hemp-based therapeutic applications [87].

### 5.4. Cannabinol (CBN)

Cannabinol (CBN) is a cannabinoid with mild psychoactive effects, which is a breakdown product of tetrahydrocannabinol (THC) through exposure to air over time. It is naturally found in old cannabis or hemp. However, CBN, unlike THC, has very minor psychoactive effects, and it might be used therapeutically without causing a high or a drop in mental ability. CBN mainly binds to CB1 and CB2 receptors of the endocannabinoid system, thereby modulating sleep, pain and immune functions [88].

Studies on animals and in vitro show that CBN has sedative, anti-inflammatory, antibacterial and neuroprotective effects. It has been found to induce sleep and relaxation, lower inflammation and protect nerve cells in animal models. Moreover, CBN may be an appetite stimulant and could be useful for the management of chronic pain disorders, which makes it a good option for health and medical products [89].

In general, fresh hemp contains only trace amounts of CBN which is formed when THC oxidizes during storage. Generally, full-spectrum hemp extracts and oils carry not only CBN but also other cannabinoids, which collectively bring about the entourage effect and thus enhance the therapeutic effect. Current studies confirm that CBN contributes to the improvement of sleep, topical skin products and anti-inflammatory drugs; therefore, CBN’s increasing role in the manufacture of hemp-based medical and health products is stressed.

### 5.5. Terpenes and Flavonoids

**Figure 5:**
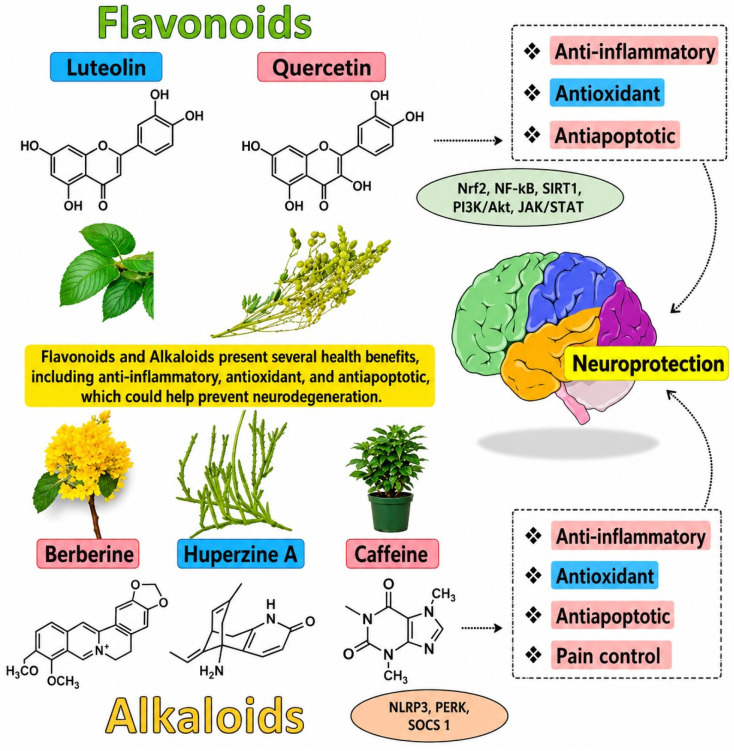
Terpenes and flavonoids [91].

Terpenes and flavonoids are chemicals that hemp (*Cannabis sativa* L.) produces naturally (Figure 5). They play a major role in determining the plant’s scent, taste and the healing effects of the product. Terpenes are a type of volatile organic compound responsible for the characteristic scent of hemp and include myrcene, limonene, linalool, caryophyllene and pinene. These substances have various biological functions such as anti-inflammatory, pain-relieving, antimicrobial, anxiolytic and antioxidant properties. They can influence how cannabinoids work through the entourage effect, thereby increasing the overall effectiveness of cannabinoids [90].

Flavonoids are polyphenols widely present in the flowers, leaves and seeds of hemp alongside cannflavins A, B and C as well as quercetin, kaempferol and luteolin. They account for the colour of the plant and have very potent antioxidant, anti-inflammatory and neuroprotective effects. Flavonoids may improve vascular health, enhance immune support and result in synergistic effects when administered with cannabinoids and terpenes in full-spectrum extracts [92].

Both terpenes and flavonoids are indispensable to the medical and health functionalities of hemp and can be considered as the natural allies of cannabinoids such as CBD, CBG, CBC and CBN. These compounds found in whole plant extracts not only help in better bioavailability but also assist in the modulation of effects and the alleviation of side effects due to the use of isolated compounds [93]. Profiling these secondary metabolites could be the key to getting the maximum benefit from hemp products in the form of therapeutics, cosmetics and food.

## 6. The Entourage Effect

**Figure 6:**
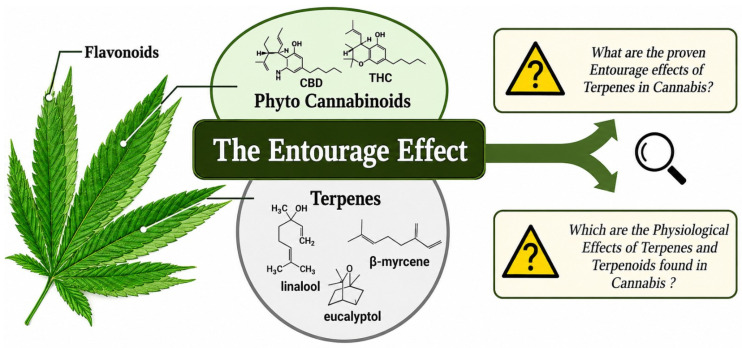
The entourage effect [95].

The entourage effect is the term used to describe the combined action of cannabinoids, terpenes, flavonoids and other bioactive compounds in hemp and cannabis that together increase the therapeutic potential of the plants. Instead of each compound going alone, they interact with each other to locally modulate the pharmacological effects, enhance bioavailability and minimize side effects (Figure 6). The whole idea behind this concept is that by using whole plant extracts you get more therapeutic benefits than you would from just one isolated compound like pure cannabidiol (CBD) [94].

Cannabinoids such as cannabidiol CBD, cannabigerol (CBG), cannabinol (CBN), and cannabichromene (CBC) act on the endocannabinoid system by altering cannabinoid receptor activity, affecting enzymes and regulating the release of neurotransmitters [96]. Terpenes like myrcene, limonene, linalool and caryophyllene not only give aroma and flavour but also have anti-inflammatory, analgesic, anxiolytic and antimicrobial properties [97].

Flavonoids add to the antioxidant and neuroprotective effects, thus potentially amplifying the therapeutic value. Both preclinical and clinical data suggest that full-spectrum hemp extracts might offer superior benefits in alleviating pain, inflammatory conditions, anxiety, seizures and neurodegenerative disorders compared to single cannabinoids. The synergistic effect of various phytochemicals can lead to an increase in receptor binding affinity, help compound transport across cellular membranes and alleviate tolerance development. Moreover, the entourage effect can result in the use of less drugs, thereby lowering the chances of undesirable effects [98].

Even though there is increasing evidence for this phenomenon, the mechanisms of the entourage effect are still not fully understood. Differences in the chemical makeup of the plants, the ways of extraction and the processes of making formulations can all greatly change the synergistic effects. To confirm these effects and to devise formulations for therapy at their best, more clinical trials with standardization as well as pharmacokinetic studies are needed. It is thus crucial to comprehend the entourage effect for the progress of safe, efficacious and scientifically backed hemp-derived medicinal products.

## 7. Safety, Toxicity, and Drug Interactions

Hemp and its derivatives, especially the non-psychoactive compounds like CBD, CBG, CBC and CBN, are mostly safe and suitable for human consumption and topical application [99]. Clinical trials and toxicological assessments show that CBD is safe for use, and the most common side effects that have been reported are mild and short-lived, such as tiredness, stomach discomfort and changes in appetite [100]. Chronic studies also show that there is little chance of dependence or of getting high when using hemp-derived products with very low THC. Besides general safety, there are some drug-related interaction issues that should not be overlooked [101]. Cannabinoids can influence cytochrome P450 enzymes which metabolize most pharmaceutical drugs, and this can change the drug’s plasma concentration and its performance as in the case of drugs such as blood thinners, anticonvulsants, antidepressants and immunosuppressants. Hence, anyone on a prescription drug should get medical advice before using hemp-derived products. Toxicity is usually low especially for the oral consumption of hemp extracts and topical uses, but overdoses or unregulated products can be a problem if there are contaminants, leftover solvents or misleading cannabinoid levels. Therefore, it is always advisable that pregnant and breastfeeding women, kids and those with liver or kidney problems be very cautious. To help ensure the safe use of hemp products in both the medical and consumer worlds, regulatory frameworks, quality assurance and standardization of dosages are essential.

## 8. Economic and Environmental Impact

### 8.1. Market Trends and Value Chains

The popularity of hemp as a crop with great potential both economically and environmentally has been gaining momentum. This has been mainly fueled by the rising global demand for sustainable products made from textiles, building materials, food, cosmetics and wellness sectors. As a result, the worldwide hemp market has been growing at an unprecedented rate. The main factors attributing to market expansion include increasing consumer awareness of the benefits of plant-based, eco-friendly products and regulatory reforms that have been supportive of the industry in many countries. In addition to the farm level, the entire hemp industry value chain, including processing, extraction, manufacturing and distribution, opens avenues for job creation in rural areas, entrepreneurship and industrial innovation. On the economic front, hemp is a pretty remarkable crop in that it can yield multiple products and hence multiple income streams from just one harvest. The seeds, fibres and core can all be turned into foods, bioplastics, textiles, paper and biofuels while the cannabinoids and extracts can be turned into high-end medicinal and wellness products. A waste-free and profitable operation can be achieved through the integrated processing and complete utilization of the hemp plant. Apart from this, the crop’s intrinsic qualities such as its adaptability, quick maturity and low need for agrochemicals make it a very attractive investment option economically compared with other traditional cash crops [102].

**Figure 7:**
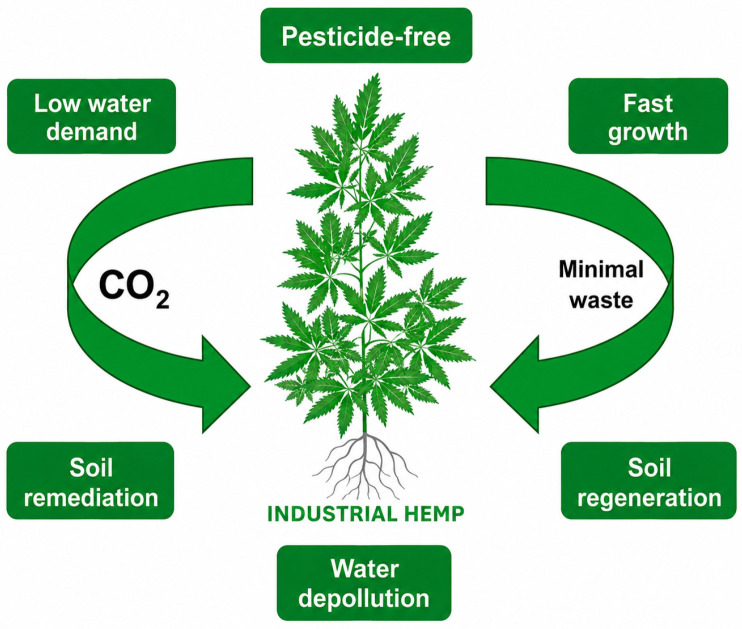
Environmental and sustainability benefits [102].

The sustainability of hemp is depicted in Figure 7, showing that it has a notably low water demand, it is grown without pesticides, it is fast growing, it can remediate water and soil and it is also capable of carbon sequestration. From an environmental angle, hemp farming is a green solution on multiple fronts: it absorbs CO_2_, cleans and revitalizes the soil, keeps the soil from washing away and lessens the need for chemical fertilizers and pesticides. Products made from hemp like bioplastics, textiles and construction composites are a great fit for circular economy frameworks as they are renewable, biodegradable and energy-saving alternatives to traditional materials. Supported by the combination of robust market expansion, versatile supply chains and beneficial environmental impacts, hemp will become a key crop for sustainable growth, bringing in both economic profits and ecological advantages.

### 8.2. Employment and Rural Development

There are great prospects for job creation and rural economic development to come through hemp cultivation and processing. As a multipurpose plant, different phases of the value chain of a hemp business, such as farming, gathering, fibre processing, oil extraction, product manufacturing and distribution, provide work. This diverse range of activities can supply both skilled and unskilled job opportunities, thus supporting the lifestyles of those living in the countryside and eventually contributing to the alleviation of poverty [103]. Hemp’s ability to produce good yields with minimal investments and tolerate various soil types and its fast growth cycle make it a crop that suits farmers operating at the scale of smallholdings or for those living hand-to-mouth. By growing hemp, rural communities can diversify their agricultural income sources, thus lowering dependence on traditional crops and increasing their level of resilience against market fluctuations.

Besides that, the manufacture and value addition of hemp products such as clothes, foodstuffs and medicines can increase the number of small and medium-sized enterprises at a local level, which in turn will lead to entrepreneurship and local economic growth. Hemp farming encourages environmentally friendly land use methods such as natural soil fertility improvement, crop rotation and less reliance on chemicals, which supports rural creatures and in the long run land productivity. Adding hemp to the local economy is consistent with the development policies at the national and regional levels which target sustainable agriculture, industrial diversification and the creation of green jobs [104].

The hemp sector has the potential to transform rural livelihoods by providing economic opportunities, enhancing food and income security and promoting environmentally sustainable development.

### 8.3. Environmental Sustainability and Carbon Sequestration

Hemp farming is one of the biggest contributors to environmental sustainability since hemp grows fast, produces lots of biomass and requires few chemicals from the farm. In contrast to traditional crops, hemp barely needs pesticides and fertilizers, which consequently decreases the chemical runoff and the damage to the soil. The roots of the plant being deep and strong, the earth’s structure gets better, soil erosion is prevented and the water-holding capacity is increased, all of which help to keep the productivity of agriculture at a good level and the health of the ecosystem intact for a long time [105].

The biggest environmental advantage of hemp is that it can soak up carbon from the air, which is the main cause of global warming. Hemp through its green leaves takes in a lot of carbon dioxide from the air and puts most of it in the fibres of the stem and the woody core (hurds). If hemp is made into long-lasting products like clothes, bioplastics, building materials such as hempcrete, then the carbon that has been absorbed and stored in the plant can stay there for long periods, thus helping to solve the problem of climate change [106]. Hemp has other uses like cleaning up the environment (phytoremediation) by getting rid of heavy metals and other chemicals. By allowing it to be a part of the cycle of growing different kinds of plants, biodiversity gets a boost, and the land is managed at a sustainable level. The features of the hemp plant make it a climatic smart crop that can greatly help in the principles of the circular economy, lessen human footprints on the environment, and provide a renewable, low-impact way of meeting industrial and consumer needs.

## 9. Challenges and Limitations

### 9.1. Legal and Policy Barriers

Despite the growing awareness of hemp as a sustainable source of materials and an economically valuable crop, legal and regulatory barriers are still very significant obstacles to its general adoption. Growing and marketing hemp can be highly regulated activities in many countries because of its closeness to the psychoactive cannabis varieties. The requirements for obtaining a licence, the very low allowed THC content and the complicated procedures for getting approval often prevent farmers, processors and manufacturers from getting into the hemp market. On the other hand, discrepancies in regulations among regions and countries make international trade more complicated, thus affecting the import and export of hemp seeds, fibres, extracts and finished products [107].

The absence of standardized testing procedures and certification schemes for THC content, cannabinoid profiles, as well as product safety lowers the trust of consumers and puts a brake on the development of the market. Furthermore, there still exist policy blanks and slow regulatory responses to industrial hemp’s low THC, non-psychoactive varieties, which limit innovation, investment and research initiatives. It is essential that the legal and policy barriers be handled if we want to have access to the full economic, therapeutic and environmental potential of hemp. Efficient licencing, clear differentiation between hemp and psychoactive cannabis, harmonized quality standards and government policies that are supportive are the main factors that can help safe cultivation, processing and commercialization to be done [108].

Such changes in the regulations would give farmers, industries and researchers the opportunity to create hemp-based merchandise in a timely manner, at the same time ensuring public safety and conforming to international standards.

### 9.2. Technical and Infrastructure Constraints

Technical and infrastructure constraints are the main barriers for the successful cultivation, processing and commercialization of hemp. Efficient hemp production is dependent on the availability of a set of machines for different stages like sowing, harvesting, retting, decortication and fibre extraction. Workers do not only work in hard conditions, but their production capacity is also lowered. Thus, the quality of the product will be significantly decreased, and the price of the product will rise due to the fewer operational costs of a large-scale unmechanized production [109].

The processing infrastructure for value-added products such as fabrics, bioplastics, food supplements and cannabinoids is at its infancy stage in many countries. A lack of proper hemp extraction and analysis units along with product development facilities set barriers to producing certified and top-notch-quality hemp products. The problem of post-harvest handling, storage and transportation is also serious enough to result in a drop in biomass quality, cannabinoid content and the quality of fibres produced from the hemp plant [110].

Agronomic knowledge deficiencies, strain selection and crop management also narrow down technical ability. Farmers who have small holdings and new businesses may not have developed their capacity and this leads to less output and a lack of uniformity in the quality of the product. To deal with such a situation, it is necessary to make investments in mechanization, processing infrastructure, research and extension services. Large-scale cultivation, industrial processing and producing competitive, high-quality hemp-based products require the establishment of reliable supply chains and training programs.

### 9.3. Market Acceptance and Competition

Market acceptance continues to be a major obstacle for hemp-based products on account of consumer perceptions, lack of knowledge and cannabis associations. Due to misunderstandings of the legality, safety and psychoactive aspects of such products, many consumers and enterprises remain reluctant toward hemp-derived products. Such ignorance might limit hemp’s application in clothing, nutraceuticals, personal care products and food sectors, even if the products comply with the regulatory and quality standards [111].

The rivalry from highly entrenched traditional materials and synthetic alternatives is yet another significant hurdle. Taking textiles for instance, the fibres of hemp must compete with cotton and polyester, whereas plastics made from hemp must face the challenge of cheap petroleum-based polymers. In the food and nutraceutical markets, products from hemp are competing with other plant-based proteins, oils and dietary supplements which already have an established supply chain and consumer loyalty [112].

It takes a concerted marketing campaign, educational efforts and branding that highlights hemp’s environmental, economic and health advantages to break through market acceptance and rivalry. Through certification, quality control and research-based assertions, the brand can showcase the effectiveness, safety and environmental friendliness of their products which in turn can boost consumer trust. Furthermore, government support, industry partnerships and the funding of creative product development can help the products derived from hemp increase their market share and boost their competitiveness in both the global and local markets.

## 10. Prospects and Innovations

### 10.1. Advances in Hemp Biotechnology

**Figure 8:**
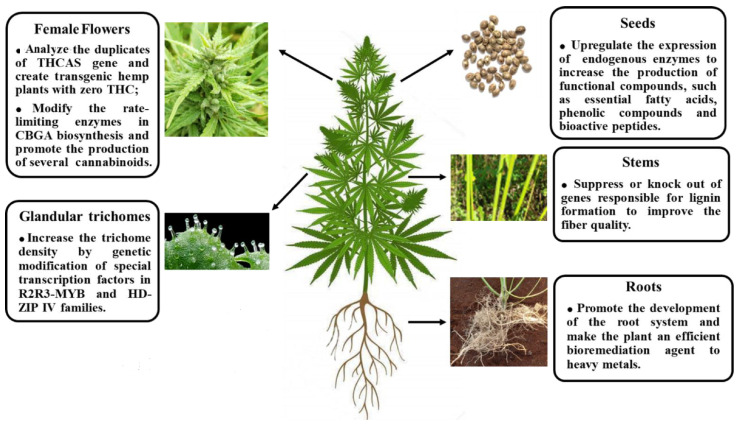
Gene editing can help to fulfil the potential usefulness of Cannabis in multiple fields, including bioenergy, textiles, food, and ecological restoration [115].

Biotechnological advances in hemp have greatly impacted the cultivation, productivity, and industrial uses of Cannabis sativa. Through selective breeding, marker-assisted selection, and genomic mapping, genetic improvement has allowed the creation of high-yielding (Figure 8), low-THC and cannabinoid-rich varieties suitable for different industrial and therapeutic applications [113].

Such developments give manufacturers the opportunity to maximize fibre quality, seed composition and cannabinoid profiles while staying within the legal THC limits. Increasingly, tissue culture and micropropagation methods are being used to produce rapid disease-free, genetically uniform planting material. These methods limit the use of seeds from traditional plant sources, speed up the propagation of top-performing cultivars and guarantee consistent chemical profiles throughout crops [50].

Furthermore, progress in CRISPR/Cas9 and other gene-editing technologies might allow for very fine alterations in the cannabinoid biosynthetic pathways, terpene production, as well as the stress tolerance of hemp. Biotechnological methods also concern the ones that can be used after harvesting to improve the processing and extraction of products. The application of enzymatic retting, fully optimized decortication, as well as extraction methods without the use of solvents brings about not only efficiency, but also the yield and the sustainability of the production of fibres, oils and cannabinoids [114].

Nanotechnology is becoming a factor in the production of hemp-based goods resulting in the enhanced bioavailability of cannabinoids as well as their use in advanced materials and medical delivery systems.

Besides boosting the economic and therapeutic value of hemp, these biotechnological advances also contribute to environmental sustainability, uniform product quality and industrial scalability. Further investigation and development in this area are essential for harnessing the full potential of hemp as a versatile crop in the 21st century.

### 10.2. Emerging Therapeutic Products

**Figure 9:**
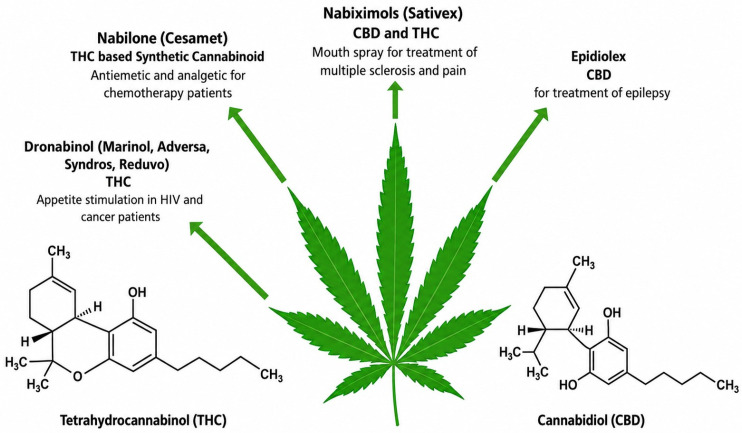
Cannabis as a source of approved drugs [117].

Hemp’s potential to serve as medicine has brought about the creation of a diverse array of new products focused on handling pain, inflammation, neurological issues, and mental health conditions. Cannabidiol (CBD), cannabigerol (CBG), cannabichromene (CBC), and other non-psychoactive cannabinoids are being put into oils, tinctures, capsules, topicals, and transdermal patches more (Figure 9). The products have anti-inflammatory, analgesic, anxiolytic and neuroprotective effects without the psychoactive effects of the hemp compounds, thus offering a safe and accessible choice for patients and wellness consumers [116].

**Figure 10:**
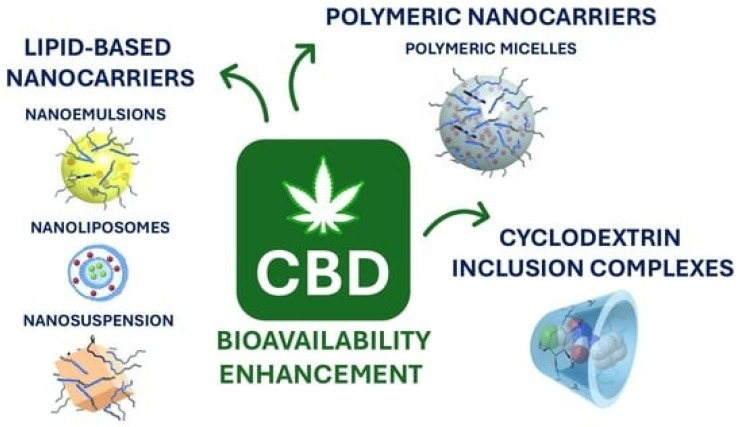
Innovative strategies to enhance the bioavailability of cannabidiol (CBD) [119].

Technological progress in extraction, purification and formulation processes has made it possible to develop full-spectrum and broad-spectrum hemp products that use the combined effect of different compounds. New delivery methods based on nanotechnology, liposomal formulas, and water-soluble cannabinoid forms significantly increase the bioavailability of cannabinoids, as represented in Figure 10, and the resultant absorption and therapeutic efficacy. There is also an increasing demand for functional foods, nutraceuticals, and beverages with cannabis derivatives, which help the consumers to include cannabis as a part of their everyday wellness in a more trusted and comfortable way [118].

In addition, products extracted from hemp are studied as potential vehicles for the delivery of therapeutics through the skin and for dermatological purposes. Examples include creams, balms, and serums that are designed to relieve pain, reduce inflammation, and promote skin health. Research at both clinical and preclinical levels is helping to generate more data on the proper dosing, pharmacokinetics, and synergistic interactions, thus setting the stage for therapeutic applications grounded in scientific evidence. These developments underscore the increasing opportunities for hemp-derived therapeutic products to address a wide range of health issues, at the same time enabling the use of safe, non-addictive, and plant-based alternatives to traditional pharmaceuticals [120].

### 10.3. Industrial Diversification Opportunities

Hemp is such a versatile crop that it offers great potential for industrial development in various sectors. This allows the use of the crop for sustainable, high-value products and at the same time, it is a way to reduce dependency on traditional raw materials. In fact, the applications of the crop are so wide-ranging that industries can go from textiles, bio-composites, and construction materials to food and nutraceuticals, cosmetics, bioplastics, biofuels, and pharmaceuticals, thereby creating new product lines and revenue streams. Moreover, the fact that it grows fast, requires little use of agrochemicals, and can even absorb carbon makes it an excellent choice of renewable resource.

**Figure 11:**
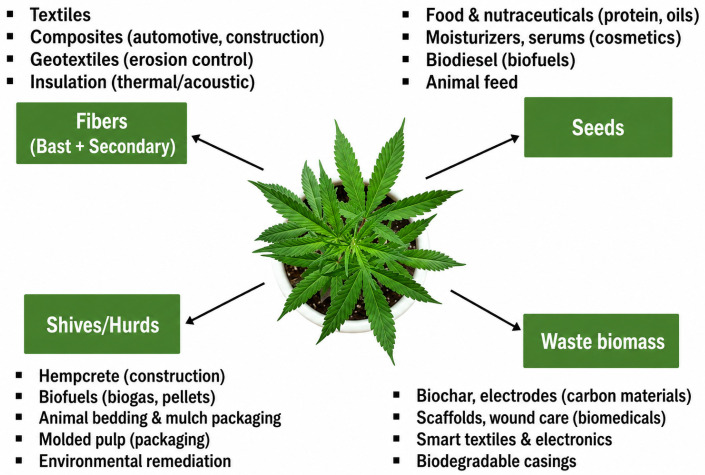
Hemp-based product applications [102].

Hemp-based materials such as hempcrete, insulation panels and fibre-reinforced composites serve as light, strong, and green alternatives to traditional building materials in the construction sector. In the manufacturing industry, automotive parts, consumer goods, and packaging increasingly contain hemp fibres and bioplastics, which are in line with circular economy concepts (Figure 11).

The food and nutraceutical industries use hemp seeds, oils, and extracts to provide the plant-based protein, essential fatty acids, and health-promoting compounds that the consumers who are health-conscious demand. In addition to that, hemp presents an avenue for small and medium-sized enterprises (SMEs) and local businesses to create value-added products ranging from farmer-level textiles and toiletries to specialized cannabinoid formulations. Efficiently embedding hemp in the current industrial value chains can be a catalyst for sustainable production, innovation, and the creation of jobs, thus satisfying the global demand for green and functional products. Taking advantage of such diversification prospects calls for the funding of processing facilities, R&D, and regulatory frameworks to guarantee product quality, uniformity, and competitiveness in the market [71].

## 11. Gaps in the Existing Literature

### 11.1. Under-Researched Applications

Extensive research has been done on the fibre, seed, and cannabidiol (CBD) components of hemp, but there are still numerous possible applications that have not been deeply explored. For example, minor cannabinoids like cannabigerol (CBG), cannabichromene (CBC), and cannabinol (CBN) possess promising therapeutic properties; however, clinical trials remain scarce to confirm their effectiveness and safety. On the other hand, the combined effects of cannabinoids, terpenes, and flavonoids, known as the entourage effect, are hardly studied; thus, the optimization of full-spectrum formulations for medical and wellness uses are still a matter of speculation [121].

Moreover, hemp industrial applications beyond textile and construction, like hemp bioplastics, advanced composites, biofuels, and valuable nutraceutical products, are not thoroughly researched. There is a lack of comprehensive studies addressing material performance over time, scalability, and environmental footprint, which hampers their commercial and industrial applications. Additionally, the use of hemp in circular economy frameworks, sustainable agriculture, and environmental remediation remains largely theoretical without much empirical evidence to support it [122].

It is vitally important to cover these research areas to utilize hemp completely as a multipurpose crop. Thorough investigations into diversified uses can lead to product innovation, policymaking, and overall economic, therapeutic, and environmental benefits of hemp-based solutions.

### 11.2. Methodological Limitations

Current research on hemp (Cannabis sativa) is still limited in certain aspects of methodology which makes the findings less reliable, comparable and generalizable. For example, most studies are limited to a small number of samples, single cultivars or only one geographical location; thus, they cannot provide evidence for different environmental and agronomic conditions. In addition, a wide range of extraction methods, analytical techniques, and experimental protocols used in studies further makes cross-study comparisons difficult, especially in terms of measuring cannabinoid, terpene, and flavonoid contents [123].

In the case of therapeutic research, preclinical studies predominantly use animal models or in vitro systems that might not be very representative of human physiology or clinical outcomes. One can hardly find standardized dosing regimens, pharmacokinetic analyses and long-term safety assessments in such studies which contributes to the gap between the lab and the medical world. Also, industrial research studies on the quality of fibres, composite materials, bioplastics, and hempcrete production usually overlook the fact that different processing methods, post-harvest handling, and materials being subjected to outdoor conditions significantly influence the final material performance [124].

Such methodological shortcomings can be rectified by implementing standardized protocols, having larger and more diverse study populations, and using interdisciplinary research approaches that combine agronomy, chemistry, pharmacology, and engineering. With enhanced scientific rigour, hemp research will benefit from reproducibility, reliability, and greater impact, thus becoming a flagship crop for industrial, medicinal and environmental applications.

## 12. Recommendations

### 12.1. Research and Development Priorities

Hemp (*Cannabis sativa*) can be a very diverse crop offering various products. However, to unlock that potential, there is a need for concerted research and development efforts in genetic improvement, cultivation, processing, and therapeutic research. Work on developing high-yield, low-THC varieties with ideal cannabinoid, terpene and fibre profiles should be regarded as the most important. It is considered that the use of biotechnological tools such as genome editing, tissue culture and micropropagation could be other means to the productivity, standardization/uniformity and disease resistance of the crop. There is a need for more research to adjust hemp crop farming practices more accurately and come up with systems that are friendly to the environment. By carrying out various experiments including the effects of different types of soil management, water-use efficiency, integrated pest control and crop rotation, not only the production but also the environment benefit could be maximized. Significant upgrading of the tech for the processing of hemp fibre, seeds, cannabinoids and other secondary metabolites is needed to achieve better product quality, larger scale and greater economic feasibility. Employing nanotechnology, enzymatic treatment and solventless extraction might be some areas where hemp products could have breakthroughs.

Among therapeutic research priorities are the clinical cannabinoid trials conducted with appropriate designs that investigate the efficacy, safety, dosage and pharmacokinetics of cannabinoids other than CBD and studies on the entourage effect and the synergistic interactions of cannabinoids, terpenes and flavonoids. Besides laboratory work, social and ethical implications along with environmental impact studies will be a crucial part of the overall research agenda. These may cover comprehensive assessments, the potential for carbon sequestration, the use of plants to clean pollutants, market studies and the optimization of the value chain. Strengthening R&D in these areas will support evidence-based policymaking, product innovation and the sustainable commercialization of hemp globally.

### 12.2. Policy Reforms

Sound policy reforms are the key to unleashing the enormous economic, industrial, and medicinal potential of hemp (*Cannabis sativa*). Due to the historical conflation of hemp with psychoactive cannabis, current regulations in many countries are still quite restrictive, which means licencing becomes a complicated process and there are very strict THC limits, and researchers and growers have limited access to the facilities. It is very important for there to be straightforward regulations that remove any ambiguity between industrial hemp and the psychoactive varieties so that it is possible to attract investment, innovate and thus grow the market.

It is the role of policymakers to lay down uniform rules for cultivation, processing, product safety, quality assurance and labelling. If issuance of permits were harmonized on the regional and international level, trade would be easier both at home and beyond borders, the costs of compliance would decrease and the consumers’ trust would be guaranteed. Policies such as the granting of subsidies, tax benefits, and research and infrastructure development which would provide more opportunities to the entrepreneurs would help rural areas to develop and the industries to diversify.

If hemp is integrated into national strategies for sustainable farming, circular economy and climate change mitigation, it can bring about green benefits while elevating the economic gains as well. There is also a need for awareness campaigns and skill development programs that will not only help the farmers but also the industry players and consumers differentiate between the products and recognize the benefits as well as follow the law and implement safe practices. Comprehensive policy reforms will open the door for the sustainable expansion of the hemp industry and lead to a thriving, innovative and globally competitive sector.

### 12.3. Industry-Capacity Building

Increasing the industrial capacity to manufacture is one of the main means through which we can sustainably develop and deeply penetrate the market of hemp (*Cannabis sativa*) in various sectors. It is not enough just to go for the best quality hemp fibres, seeds, oils and cannabinoids without also considering the necessity for a state-of-the-art investment in facilities such as cultivation equipment, processing factories, extraction units, and laboratories for quality testing. Efficient supply chains and packaging at harvest can be the determinant of a product retaining less loss, staying fresh, and being competitive in the market.

Human resource development through training and educational programs for farmers, processors, and product developers is a must to raise the level of best practices in cultivation, harvesting, processing, and formulation. Apart from that, technical workshops, extension services, and certification programs can lead to the improvement of knowledge transfer, the adoption of innovation, and thus the increase in operational efficiency along the whole hemp chain. On the one hand, academic institutions and research centres as well as industry stakeholders can create a joint effort through consultation that will allow the facilitation of technology transfer, the support of R&D, and the emergence of value-added products in the market.

Capacity-building programs can primarily boost small and medium-sized enterprises (SMEs) and rural industries in different ways such as through promoting entrepreneurship, generating job opportunities, and facilitating the local economic development. Hence, by mobilizing human capital, upgrading technological expertise, and increasing institutional backings, capacity enrichment of the industry will contribute to the competitiveness, sustainability, and scalability of the hemp sector, so that it can satisfy the rising world demand while at the same time providing economic, therapeutic, and environmental advantages.

## 13. Conclusions: Summary of Key Findings

Through this review, the author demonstrates the various ways hemp (*Cannabis sativa*) can be an environmentally friendly and highly adaptable crop with great potential for industrial, therapeutic, environmental and economic uses. From an industrial perspective, hemp can yield fibres, seeds and biomass of superior quality for use in textiles, construction materials, bioplastics, automotive composites and packaging, thus providing a renewable source for the replacement of traditional resources.

On a therapeutic level, cannabinoids such as CBD, CBG, CBC and CBN in combination with terpenes and flavonoids have been found to have anti-inflammatory, analgesic, neuroprotective and antioxidant effects, thereby opening a broad range of possibilities for functional foods, nutraceuticals and cosmetic products. As a result of hemp farming, various aspects of environmental sustainability are achieved; hemp helps the soil to be more fertile, takes in carbon, can be used in phytoremediation and encourages the practices of the circular economy.

On the economic front, the benefits are a diverse market, employment in the rural areas and the continuous creation of value-added products through global and local supply chains. Some impediments exist, however, among which are regulatory issues, the lack of technical know-how and necessary infrastructure, the market not being fully ready and scientific research being limited especially when it comes to minor cannabinoids, product standardization and processing on a large scale. Innovative approaches in biotechnology, product formulation and industrial applications, along with well planned research, changes to the laws and capacity building can help overcome these challenges.

Hemp is a high-value multipurpose crop which has great potential for sustainable development, industrial diversification and public health if research, regulation and industry support are adequately coordinated.

## Figures and Tables

**Table 1:** Hemp pest management.

Pest/Disease	Management Approach	Notes for Industrial/Therapeutic Hemp
Aphids	Biological (ladybugs), neem oil, pruning	Avoid chemical residues on flowers
Spider mites	Predatory mites, neem oil, good airflow	Critical to maintain cannabinoid quality
Caterpillars	Handpicking, traps	Small-scale mechanical control effective
Powdery mildew	Resistant varieties, pruning, sulfur sprays	Reduces quality of fibre and cannabinoid content
Botrytis (grey mould)	Pruning, airflow, biological fungicides	Severe impact on flower quality; must prevent

## Data Availability

Further inquiries can be directed to the corresponding author.
